# Tumor infiltrating lymphocyte stratification of prognostic staging of early-stage triple negative breast cancer

**DOI:** 10.1038/s41523-021-00362-1

**Published:** 2022-01-11

**Authors:** Sherene Loi, Roberto Salgado, Sylvia Adams, Giancarlo Pruneri, Prudence A. Francis, Magali Lacroix-Triki, Heikki Joensuu, Maria Vittoria Dieci, Sunil Badve, Sandra Demaria, Robert Gray, Elisabetta Munzone, Damien Drubay, Jerome Lemonnier, Christos Sotiriou, Pirkko Liisa Kellokumpu-Lehtinen, Andrea Vingiani, Kathryn Gray, Fabrice André, Carsten Denkert, Martine Piccart, Elvire Roblin, Stefan Michiels

**Affiliations:** 1 Peter MacCallum Cancer Centre, Melbourne, VIC Australia; 2 Sir Peter MacCallum Cancer Department of Oncology, University of Melbourne, Melbourne, VIC Australia; 3 Department of Pathology, GZA-ZNA, Antwerp, Belgium; 4 New York University Langone Health, Perlmutter Cancer Center, New York, NY USA; 5 Fondazione Istituto di Ricovero e Cura a Carattere Scientifico-Isituto Nazionale dei Tumori, Universita degli Studi di Milano, Milan, Italy; 6 Gustave Roussy, Université Paris-Saclay, Villejuif, France; 7 Helsinki University Hospital and University of Helsinki, Helsinki, Finland; 8 Department of Surgery, Oncology and Gastroenterology, University of Padua, Padua, Italy; 9 Medical Oncology 2, Veneto Institute of Oncology IOV - IRCCS, Padua, Italy; 10 Indiana University, Indianapolis, IN USA; 11 Weill-Cornell Medicine, New York, NY USA; 12 Dana-Farber Cancer Institute, Boston, MA USA; 13 Division of Medical Senology, European Institute of Oncology, IRCCS, Milan, Italy; 14 Service de Biostatistique et d’Epidémiologie, Gustave Roussy, Oncostat U1018, Inserm, Paris-Saclay University, labeled Ligue Contre le Cancer, Villejuif, France; 15 R&D UNICANCER, Paris, France; 16 Institut Jules Bordet, Universite Libre de Bruxelles, Brussels, Belgium; 17 Faculty of Medicine and Health Technology Tampere University and Tampere University Hospital Tamper, Tamper, Finland; 18 Frontier Science & Technology Research Foundation, Boston, MA USA; 19 Gustave Roussy, Université Paris-Saclay, Villejuif, France; 20 Institut für Pathologie, Universitätsklinikum Marburg, Philipps-Universität Marburg, Marburg, Germany

**Keywords:** Breast cancer, Prognostic markers

## Abstract

The importance of integrating biomarkers into the TNM staging has been emphasized in the 8^th^ Edition of the American Joint Committee on Cancer (AJCC) Staging system. In a pooled analysis of 2148 TNBC-patients in the adjuvant setting, TILs are found to strongly up and downstage traditional pathological-staging in the Pathological and Clinical Prognostic Stage Groups from the AJJC 8^th^ edition Cancer Staging System. This suggest that clinical and research studies on TNBC should take TILs into account in addition to stage, as for example patients with stage II TNBC and high TILs have a better outcome than patients with stage I and low TILs.

## Introduction

The importance of integrating biomarkers into the TNM staging has been emphasized in the 8^th^ Edition of the American Joint Committee on Cancer (AJCC) Staging System (www.cancerstaging.org). Prognostic biomarkers are becoming increasingly important given that many low early-stage breast cancer patients have excellent outcomes with standard of care treatments. The clinical guidelines for systemic treatment in early-stage TNBC are not consistent. The evidence for the integration of biomarkers into the TNM staging system is mostly retrospective and with limited prospective data available^1^. In the case of multigene panels, their clinical validity for defining prognosis of particular subgroups of patients had been shown in several retrospective analyses and these were routinely used and incorporated into national (NCCN and ASCO) and international (ESMO, St Gallen) treatment guidelines far before prospective data became available. In the 8^th^ edition of the AJCC, histologic grading, molecular subtype (luminal, HER2-positive, and triple negative [TN]) and multigene panels/genomic signatures were included into staging assessments. Whilst pathological and clinical stage remain a valuable aspect of the staging process and are strongly prognostic, we highlight here the evidence that supports Tumor Infiltrating Lymphocytes (TILs) as a biologic biomarker which improves discrimination over prognostic pathological and clinical staging for early stage TNBC.

**Fig. 1:**
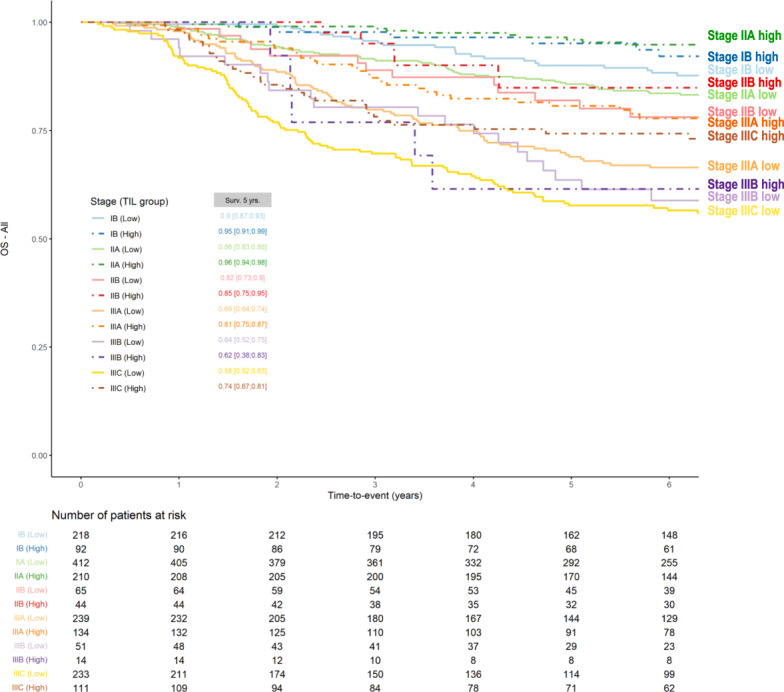
Kaplan–Meier curves of overall survival of triple-negative breast cancer patients treated with anthracycline-based chemotherapy with or without taxanes, according to pathological prognostic stage and TILs. Pathological Prognostic Stage is assigned stage for patients who have surgical resection as the initial treatment of their cancer before receipt of any systemic or radiation therapy. It is based on clinical information, biomarker data, and findings from surgery and resected tissue. The 5-year estimated overall survival values (5-Yr OS) are provided together with bootstrap confidence intervals.

**Fig. 2:**
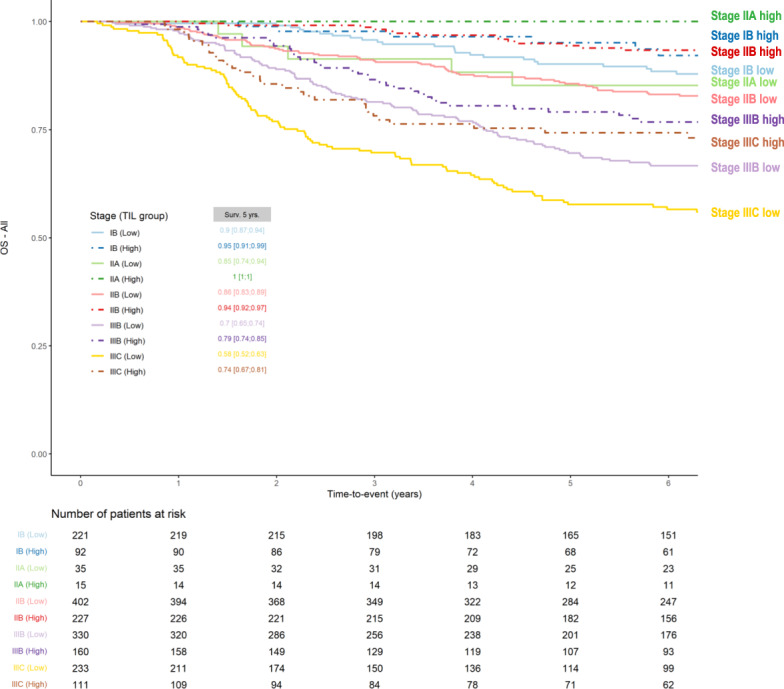
Kaplan–Meier curves of overall survival of triple-negative breast cancer patients treated with anthracycline-based chemotherapy with or without taxanes, according to clinical prognostic stage and TILs. Clinical prognostic stage assigned stage for all patients based on history, physical examination, imaging studies performed (but not required) and relevant biopsies. Clinical prognostic stage is determined by T, N, M, tumor grade, as well as subtype information using human epidermal growth factor receptor (HER2), estrogen receptor (ER), and progesterone receptor (PR)- status. Supplementary Tables 1 and 2.

A recent pooled analysis provides the relevant work for integrating TIL quantity into clinical staging for early-stage TNBC (JCO 2019)^2^. These data were from 2148 patients enrolled onto eight prospective clinical trials as well as one large institutional cohort. All patients had received anthracyclines with or without taxanes based regimens in the adjuvant setting. The median follow-up was 6.6 years. In the current study, using the Pathological and Clinical Prognostic Stage Groups from the AJJC 8th edition Cancer Staging Manual (for definitions hereof, see footnote*), three endpoints were calculated for each Stage group: invasive disease-free survival (iDFS), distant disease-free survival (D-DFS) as well as overall survival (OS). At a cut-point of 30% or more, TIL scores are found to strongly up and downstage traditional pathological-staging in this large data set (Fig. 1, Supp Table 1). This suggest that clinical and research studies on TNBC should take TILs into account in addition to stage, as patients with stage II TNBC and high TILs have a better outcome than patients with stage I and low TILs. Importantly, histological grade is not prognostic in this pooled analysis. At the established prognostic cut-off of 30%, trained pathologists have a high pairwise concordance rate up to 0.93 using both core-biopsies as well as full sections, indicating that TILs are a reliable biomarker that can be used in daily practice and in research and clinical trial settings^3^. This compares favorably to the reproducibility of other commonly used morphological features in breast cancer practice^4^. Similarly, adding TILs to clinical staging is shown to up- and downstage prognostic groups which also take subtype into consideration (Fig. 2; Supp Table 2) though we note the small numbers in some of the subgroups (ie Stage IIA).

Risk assessment models meeting the AJCC criteria and included in the 8^th^ Edition were Adjuvant Online and PREDICT-Plus. We have developed a prognostic model for early-stage disease that includes age, number of positive lymph nodes, tumor size, grade, and stromal TIL percentage. This prognostic model is available on the website of the International Immuno-Oncology Biomarker Working Group (www.tilsinbreastcancer.org). This prognostic model could be used in routine patient care as well as future trial designs.

The inclusion of immune biomarkers is timely given reporting of phase III registration trials of PD-1/PD-L1 targeting agents in early-stage TNBC. In the recently reported KEYNOTE-522 study (NCT03036488)^5^, pembrolizumab, a PD-1 inhibitor, was found to significantly but moderately increase the rates of pathological complete response (pCR) and significantly improve event-free survival (EFS) when added to a neoadjuvant regimen of anthracycline, taxanes and carboplatin in patients with stage II and III TNBC. Similar magnitude of EFS improvements have been reported in smaller studies^6,7^. PD-L1 protein is known to be highly expressed on the breast cancer TIL and correlations between both markers have been observed^8^. In the KEYNOTE-522 study, patients designated as PD-L1 positive by the PD-L1 IHC 22C3 pharmdDx assay (Agilent Technologies, Carpinteria, CA, USA) were reported to have higher rates of pCR in both pembrolizumab and control arms across all TNM stages. An important treatment by PD-L1 interaction was not observed, in contrast to late-stage disease^9,10^. In any case, the increasing incorporation of PD-1 inhibition into the treatment of both early- and late stage TNBC further reinforces the need for routine evaluation of TIL in all TNBC patients, particularly given discordances in PD-L1 IHC assays. One could also speculate that patients with high expression of both immune biomarkers, i.e. those “immune enriched” could be strong candidates for future treatment optimization (for example, reduced cytotoxic chemotherapy)^11^.

## Methods

This is a follow-up analysis of the Loi et al 2019 study^2^. The study protocol of this project was approved by the local institutional review committee at Gustave Roussy in November 2014 and authorized by the French National Committee on Computing and Liberty. Identified studies were prospective randomized clinical trials or large retrospective hospital series that evaluated the prognostic associations of TILs in patients who were diagnosed with early-stage TNBC treated with anthracycline-based chemotherapy with or without taxane in the adjuvant setting using the same prespecified method for reading TILS (according to International Immuno-Oncology Biomarker Working Group guidelines). TNBC was defined by estrogen receptor (ER), progesterone receptor (PR), and HER2 negativity as outlined in Loi et al. (ref).

The prespecified primary end point was invasive disease-free survival (iDFS), defined as the date of first invasive recurrence, or second primary or death from any cause. Patients still alive without an event of interest were censored at the date of the last visit. Distant disease-free survival (D-DFS) is defined as the date of first distant recurrence or death from any cause. Patients still alive without an event of interest were censored at the date of the last visit. Overall survival (OS) was defined as the date of death from any cause. Patients still alive were censored at the date of the last visit. We used the Kaplan-Meier method to establish survival curves according to AJCC staging categories. The Kaplan–Meier estimates are provided together with bootstrap confidence intervals (1000 samples).

### Reporting summary

Further information on research design is available in the Nature Research Reporting Summary linked to this article.

## Supplementary information

Supplementary Information

Reporting Summary

## Data Availability

Trial data can be made available by contacting the individual trial investigators of the trials included in this project, pending individual sponsors approval. The corresponding author of the paper can redirect requests. A specific data transfer agreement between the individual sponsors of the trials and the researcher may be requested. This data transfer agreement details both parts responsibilities to ensure the required level of data integrity and legal and ethical obligations.
