# Supplementary material for: Tumor infiltrating lymphocyte stratification of prognostic staging of early-stage triple negative breast cancer
Source: NPJ Breast Cancer. 2022 Jan 11;8:3. doi: 10.1038/s41523-021-00362-1 (PMC8752727; doi:10.1038/s41523-021-00362-1)
Supplement: Supplementary file 1 — Supplementary Information [file 41523_2021_362_MOESM1_ESM.pdf]

Supplementary Table 1. Estimated 5-year survival probabilities according to Pathological Prognostic Stage and stratified by TILs in triple-negative breast cancer patients treated in the adjuvant setting with anthracycline-based chemotherapy with or without taxanes.

OS: overall survival, IDFS: invasive-disease free survival, D-DFS: distant disease-free survival (for definitions see Loi et al JCO 2019). The 5-year Kaplan-Meier estimates are provided together with bootstrap confidence intervals (1000 samples).

T size	Nodal status	Grade	Pathological Prognostic Stage Group	N	TIL group High ≥30% Low <30%	5-Yr OS	5-Yr iDFS	5-Yr D-DFS
T1 <2cm	N0	G1	IA	0	High			
T1	N0	G1	IA	3	Low			
T1	N0	G2	IB	6	High	0.95 [0.91;0.99]	0.86 [0.80;0.92]	0.91 [0.86;0.96]
T1	N0	G3	IB	86				
T1	N0	G2	IB	47	Low	0.90 [0.86;0.93]	0.84 [0.80;0.88]	0.88 [0.84;0.92]
T1	N0	G3	IB	171				
T1	N1 (1-3)	G1	IIA	14	High	0.96 [0.94;0.98]	0.89 [0.85;0.92]	0.94 [0.91;0.97]
T2 2-5cm	N0	G1	IIA	1				
T1	N1	G2	IIA	13				
T2	N0	G2	IIA	5				
T1	N1	G3	IIA	79				
T2	N0	G3	IIA	98				
T1	N1	G1	IIA	34	Low	0.86 [0.83;0.89]	0.76 [0.73;0.80]	0.83 [0.80;0.85]
T2	N0	G1	IIA	1				
T1	N1	G2	IIA	39				
T2	N0	G2	IIA	34				
T1	N1	G3	IIA	126				
T2	N0	G3	IIA	178				
T2	N1	G1	IIB	32	High	0.85 [0.74,0.93]	0.81 [0.71;0.91]	0.83 [0.74;0.93]
T3 >5cm	N0	G1	IIB	0				
T2	N1	G2	IIB	12				
T3	N0	G2	IIB	0				
T2	N1	G1	IIB	25	Low	0.82 [0.74;0.90]	0.71 [0.61;0.81]	0.76 [0.67;0.84]
T3	N0	G1	IIB	0				
T2	N1	G2	IIB	40				
T3	N0	G2	IIB	0				
T2	N1	G3	IIIA	103	High	0.81 [0.75;0.86]	0.75 [0.69;0.81]	0.79 [0.73;0.84]
T3	N0	G3	IIIA	5				
T1	N2 (≥4)	G1	IIIA	8				
T2	N2	G1	IIIA	17				
T3	N1	G1	IIIA	1				
T3	N2	G1	IIIA	0				
T2	N1	G3	IIIA	201	Low	0.69 [0.64;0.74]	0.60 [0.55;0.65]	0.65 [0.60;0.69]
T3	N0	G3	IIIA	12				
T1	N2	G1	IIIA	10				
T2	N2	G1	IIIA	12				
T3	N1	G1	IIIA	1				
T3	N2	G1	IIIA	3				
T1	N2	G2	IIIB	6	High	0.62 [0.38;0.83]	0.57 [0.36;0.79]	0.62 [0.39;0.85]
T2	N2	G2	IIIB	6				
T3	N1	G2	IIIB	0				
T3	N2	G2	IIIB	2				
T1	N2	G2	IIIB	15	Low	0.64 [0.52;0.74]	0.49 [0.37;0.59]	0.52 [0.41;0.64]
T2	N2	G2	IIIB	26				
T3	N1	G2	IIIB	5				
T3	N2	G2	IIIB	5				
T1	N2	G3	IIIC	34	High	0.74 [0.68;0.81]	0.67 [0.60;0.74]	0.69 [0.61;0.76]
T2	N2	G3	IIIC	65				
T3	N1	G3	IIIC	5				

T size	Nodal status	Grade	Pathological Prognostic Stage Group	N	TIL group High ≥30% Low <30%	5-Yr OS	5-Yr iDFS	5-Yr D-DFS
T3	N2	G3	IIIC	7				
T1	N2	G3	IIIC	59	Low	0.58 [0.52;0.63]	0.49 [0.44;0.55]	0.54 [0.48;0.59]
T2	N2	G3	IIIC	127				
T3	N1	G3	IIIC	13				
T3	N2	G3	IIIC	34				

Supplementary Table 2 : Estimated 5-year survival probabilities according to Pathological Prognostic Stage and stratified TILs in triple-negative breast cancer patients treated with anthracycline-based chemotherapy with taxanes in the adjuvant setting.

OS: overall survival, IDFS: invasive-disease free survival, D-DFS: distant disease-free survival (for definitions see Loi et al JCO 2019). The 5-year Kaplan-Meier estimates are provided together with bootstrap confidence intervals (1000 samples).

T size	Nodal status	Grade	Pathological Prognostic Stage Group	N	TIL group High ≥30% Low <30%	5-Yr OS	5-Yr IDFS	5-Yr D-DFS
T1 <2cm	N0	G1	IA	0	High			
T1	N0	G1	IA	2	Low			
T1	N0	G2	IB	2	High	0.86 [0.62;1]	0.80 [0.60;1]	0.86 [0.60;1]
T1	N0	G3	IB	8				
T1	N0	G2	IB	8	Low	0.92 [0.84;0.98]	0.90 [0.81;0.96]	0.93 [0.86;0.98]
T1	N0	G3	IB	42				
T1	N1 (1-3)	G1	IIA	6	High	0.98 [0.94;1]	0.96 [0.91;1]	0.98 [0.95;1]
T2 2-5cm	N0	G1	IIA	0				
T1	N1	G2	IIA	6				
T2	N0	G2	IIA	0				
T1	N1	G3	IIA	25				
T2	N0	G3	IIA	19				
T1	N1	G1	IIA	12	Low	0.85 [0.80;0.90]	0.75 [0.69;0.81]	0.82 [0.76;0.87]
T2	N0	G1	IIA	0				
T1	N1	G2	IIA	19				
T2	N0	G2	IIA	7				
T1	N1	G3	IIA	54				
T2	N0	G3	IIA	52				
T2	N1	G1	IIB	12	High	0.82 [0.65;0.94]	0.78 [0.61;0.94]	0.83 [0.66;0.95]
T3 >5cm	N0	G1	IIB	0				
T2	N1	G2	IIB	7				
T3	N0	G2	IIB	0				
T2	N1	G1	IIB	19	Low	0.79 [0.67;0.91]	0.63 [0.50;0.76]	0.68 [0.56;0.80]
T3	N0	G1	IIB	0				
T2	N1	G2	IIB	18				
T3	N0	G2	IIB	0				
T2	N1	G3	IIIA	41	High	0.83 [0.74;0.92]	0.79 [0.69;0.88]	0.83 [0.73;0.92]
T3	N0	G3	IIIA	1				
T1	N2 (≥4)	G1	IIIA	2				
T2	N2	G1	IIIA	4				
T3	N1	G1	IIIA	1				
T3	N2	G1	IIIA	0				
T2	N1	G3	IIIA	88	Low	0.68 [0.60;0.76]	0.58 [0.50;0.65]	0.63 [0.55;0.71]
T3	N0	G3	IIIA	3				
T1	N2	G1	IIIA	8				
T2	N2	G1	IIIA	7				
T3	N1	G1	IIIA	1				
T3	N2	G1	IIIA	2				
T1	N2	G2	IIIB	4	High	0.86 [0.62;1]	0.75 [0.50;1]	0.86 [0.60;1]
T2	N2	G2	IIIB	4				
T3	N1	G2	IIIB	0				
T3	N2	G2	IIIB	0				
T1	N2	G2	IIIB	6	Low	0.69 [0.54;0.84]	0.50 [0.34;0.65]	0.53 [0.37;0.69]
T2	N2	G2	IIIB	12				
T3	N1	G2	IIIB	4				
T3	N2	G2	IIIB	4				
T1	N2	G3	IIIC	14	High	0.67 [0.56;0.79]	0.56 [0.44;0.68]	0.58 [0.45;0.70]
T2	N2	G3	IIIC	22				

T size	Nodal status	Grade	Pathological Prognostic Stage Group	N	TIL group High ≥30% Low <30%	5-Yr OS	5-Yr IDFS	5-Yr D-DFS
T3	N1	G3	IIIC	4				
T3	N2	G3	IIIC	4				
T1	N2	G3	IIIC	22	Low	0.58 [0.49;0.67]	0.54 [0.45;0.63]	0.57 [0.48;0.66]
T2	N2	G3	IIIC	47				
T3	N1	G3	IIIC	8				
T3	N2	G3	IIIC	14				

Supplementary Table 2a: Estimated 5-year survival probabilities according to Clinical Prognostic Stage and stratified by TILs in triple-negative breast cancer patients treated with anthracycline-based chemotherapy with or without taxanes in the adjuvant setting.

OS: overall survival, IDFS: invasive-disease free survival, D-DFS: distant disease-free survival (for definitions see Loi et al JCO 2019). The 5-year Kaplan-Meier estimates are provided together with bootstrap confidence intervals (1000 samples).

T size	Nodal status	Grade	Clinic Prognostic Stage Group	N	TIL group High ≥30% Low <30%	5 Yr OS	5 Yr IDFS	5 Yr D-DDFS
T1 <2cm	N0	G1	IB	0	High	0.95 [0.91;0.99]	0.86 [0.80;0.92]	0.91 [0.86;0.96]
T1	N0	G2	IB	6				
T1	N0	G3	IB	86				
T1	N0	G1	IB	3	Low	0.90 [0.87;0.94]	0.84 [0.79;0.88]	0.88 [0.85;0.92]
T1	N0	G2	IB	47				
T1	N0	G3	IB	171				
T1	N1 (1-3)	G1	IIA	14	High	1 [1;1]	0.93 [0.79;1]	0.93 [0.79;1]
T2 2-5cm	N0	G1	IIA	1				
T1	N1	G1	IIA	34	Low	0.85 [0.74;0.94]	0.78 [0.64;0.88]	0.85 [0.74;0.94]
T2	N0	G1	IIA	1				
T1	N1	G2	IIB	13	High	0.94 [0.92;0.97]	0.87 [0.84;0.91]	0.93 [0.90;0.95]
T2	N0	G2	IIB	5				
T1	N1	G3	IIB	79				
T2	N0	G3	IIB	98				
T2	N1	G1	IIB	32				
T3 >5cm	N0	G1	IIB	0				
T1	N1	G2	IIB	39	Low	0.86 [0.83;0.88]	0.76 [0.72;0.79]	0.82 [0.78;0.85]
T2	N0	G2	IIB	34				
T1	N1	G3	IIB	126				
T2	N0	G3	IIB	178				
T2	N1	G1	IIB	25				
T3	N0	G1	IIB	0				
T2	N1	G2	IIIB	12	High	0.79 [0.74;0.84]	0.74 [0.68;0.80]	0.78 [0.72;0.83]
T3	N0	G2	IIIB	0				
T2	N1	G3	IIIB	103				
T3	N0	G3	IIIB	5				
T1	N2 (≥4)	G1	IIIB	8				
T2	N2	G1	IIIB	17				
T3	N1	G1	IIIB	1				
T3	N2	G1	IIIB	0				
T1	N2	G2	IIIB	6				
T2	N2	G2	IIIB	6				
T3	N1	G2	IIIB	0				
T3	N2	G2	IIIB	2				
T2	N1	G2	IIIB	40	Low	0.70 [0.65;0.73]	0.60 [0.55;0.64]	0.64 [0.50;0.69]
T3	N0	G2	IIIB	0				
T2	N1	G3	IIIB	201				
T3	N0	G3	IIIB	12				
T1	N2	G1	IIIB	10				
T2	N2	G1	IIIB	12				
T3	N1	G1	IIIB	1				
T3	N2	G1	IIIB	3				
T1	N2	G2	IIIB	15				
T2	N2	G2	IIIB	26				
T3	N1	G2	IIIB	5				
T3	N2	G2	IIIB	5				
T1	N2	G3	IIIC	34	High	0.74 [0.67;0.82]	0.67 [0.59;0.74]	0.69 [0.61;0.76]
T2	N2	G3	IIIC	65				

T size	Nodal status	Grade	Clinic Prognostic Stage Group	N	TIL group High ≥30% Low <30%	5 Yr OS	5 Yr IDFS	5 Yr D-DDFS
T3	N1	G3	IIIC	5				
T3	N2	G3	IIIC	7				
T1	N2	G3	IIIC	59	Low	0.58 [0.52;0.63]	0.49 [0.44;0.55]	0.54 [0.48;0.59]
T2	N2	G3	IIIC	127				
T3	N1	G3	IIIC	13				
T3	N2	G3	IIIC	34				

Supplementary Table 2b: Estimated 5-year survival probabilities according to Clinical Prognostic Stage and stratified by TILs in triple-negative breast cancer patients treated with anthracycline-based chemotherapy with taxanes in the adjuvant setting.

OS: overall survival, IDFS: invasive-disease free survival, D-DFS: distant disease-free survival (for definitions see Loi et al JCO 2019). The 5-year Kaplan-Meier estimates are provided together with bootstrap confidence intervals (1000 samples).

T size	Nodal status	Grade	Clinic Prognostic Stage Group	N	TIL group High ≥30% Low <30%	5 Yr OS	5 Yr IDFS	5 Yr D-DFS
T1 <2cm	N0	G1	IB	0	High	0.86 [0.62;1]	0.80 [0.60;1]	0.86 [0.60;1]
T1	N0	G2	IB	2				
T1	N0	G3	IB	8				
T1	N0	G1	IB	2	Low	0.93 [0.86;0.88]	0.87 [0.78;0.94]	0.93 [0.85;0.98]
T1	N0	G2	IB	8				
T1	N0	G3	IB	42				
T1	N1 (1-3)	G1	IIA	6	High	1 [1;1]	1 [1;1]	1 [1;1]
T2 2-5cm	N0	G1	IIA	0				
T1	N1	G1	IIA	12	Low	0.75 [0.58;0.92]	0.75 [0.50;0.92]	0.75 [0.58;0.92]
T2	N0	G1	IIA	0				
T1	N1	G2	IIB	6	High	0.96 [0.92;1]	0.93 [0.87;0.98]	0.97 [0.92;1]
T2	N0	G2	IIB	0				
T1	N1	G3	IIB	25				
T2	N0	G3	IIB	19				
T2	N1	G1	IIB	12				
T3 >5cm	N0	G1	IIB	0				
T1	N1	G2	IIB	19	Low	0.86 [0.81;0.91]	0.74 [0.67;0.80]	0.81 [0.76;0.86]
T2	N0	G2	IIB	7				
T1	N1	G3	IIB	54				
T2	N0	G3	IIB	52				
T2	N1	G1	IIB	19				
T3	N0	G1	IIB	0				
T2	N1	G2	IIIB	7	High	0.82 [0.74;0.89]	0.78 [0.70;0.86]	0.82 [0.74;0.90]
T3	N0	G2	IIIB	0				
T2	N1	G3	IIIB	41				
T3	N0	G3	IIIB	1				
T1	N2 (≥4)	G1	IIIB	2				
T2	N2	G1	IIIB	4				
T3	N1	G1	IIIB	1				
T3	N2	G1	IIIB	0				
T1	N2	G2	IIIB	4				
T2	N2	G2	IIIB	4				
T3	N1	G2	IIIB	0				
T3	N2	G2	IIIB	0				
T2	N1	G2	IIIB	18	Low	0.69 [0.62;0.75]	0.57 [0.51;0.64]	0.61 [0.54;0.68]
T3	N0	G2	IIIB	0				
T2	N1	G3	IIIB	88				
T3	N0	G3	IIIB	3				
T1	N2	G1	IIIB	8				
T2	N2	G1	IIIB	7				
T3	N1	G1	IIIB	1				
T3	N2	G1	IIIB	2				
T1	N2	G2	IIIB	6				
T2	N2	G2	IIIB	12				
T3	N1	G2	IIIB	4				
T3	N2	G2	IIIB	4				
T1	N2	G3	IIIC	14	High	0.67 [0.55;0.79]	0.58 [0.44;0.67]	0.58 [0.45;0.70]
T2	N2	G3	IIIC	22				
T3	N1	G3	IIIC	4				

**T size**	**Nodal status**	**Grade**	**Clinic Prognostic Stage Group**	**N**	**TIL group High ≥30% Low <30%**	**5 Yr OS**	**5 Yr IDFS**	**5 Yr D-DFS**
T3	N2	G3	IIIC	4				
T1	N2	G3	IIIC	22	Low	0.58 [0.48;0.67]	0.54 [0.45;0.62]	0.57 [0.48;0.66]
T2	N2	G3	IIIC	47				
T3	N1	G3	IIIC	8				
T3	N2	G3	IIIC	14				
