# Supplementary material for: Tumor infiltrating lymphocyte stratification of prognostic staging of early-stage triple negative breast cancer
Source: NPJ Breast Cancer. 2022 Jan 11;8:3. doi: 10.1038/s41523-021-00362-1 (PMC8752727; doi:10.1038/s41523-021-00362-1)
Supplement: Supplementary file 2 — Reporting Summary [file 41523_2021_362_MOESM2_ESM.pdf]

nature portfolio

Corresponding author(s): Prof. Sherene Loi

Last updated by author(s): Sep 3, 2021

Reporting Summary

Nature Portfolio wishes to improve the reproducibility of the work that we publish. This form provides structure for consistency and transparency in reporting. For further information on Nature Portfolio policies, see our Editorial Policies and the Editorial Policy Checklist.

Statistics

For all statistical analyses, confirm that the following items are present in the figure legend, table legend, main text, or Methods section.

n/a	Confirmed	
☐	☒	The exact sample size (*n*) for each experimental group/condition, given as a discrete number and unit of measurement
☒	☐	A statement on whether measurements were taken from distinct samples or whether the same sample was measured repeatedly
☒	☐	The statistical test(s) used AND whether they are one- or two-sided<br>*Only common tests should be described solely by name; describe more complex techniques in the Methods section.*
☒	☐	A description of all covariates tested
☒	☐	A description of any assumptions or corrections, such as tests of normality and adjustment for multiple comparisons
☐	☒	A full description of the statistical parameters including central tendency (e.g. means) or other basic estimates (e.g. regression coefficient) AND variation (e.g. standard deviation) or associated estimates of uncertainty (e.g. confidence intervals)
☒	☐	For null hypothesis testing, the test statistic (e.g. *F*, *t*, *r*) with confidence intervals, effect sizes, degrees of freedom and *P* value noted<br>*Give P values as exact values whenever suitable.*
☒	☐	For Bayesian analysis, information on the choice of priors and Markov chain Monte Carlo settings
☒	☐	For hierarchical and complex designs, identification of the appropriate level for tests and full reporting of outcomes
☒	☐	Estimates of effect sizes (e.g. Cohen's *d*, Pearson's *r*), indicating how they were calculated

Our web collection on statistics for biologists contains articles on many of the points above.

Software and code

Policy information about availability of computer code

Data collection	R software
Data analysis	R software

For manuscripts utilizing custom algorithms or software that are central to the research but not yet described in published literature, software must be made available to editors and reviewers. We strongly encourage code deposition in a community repository (e.g. GitHub). See the Nature Portfolio guidelines for submitting code & software for further information.

Data

Policy information about availability of data

All manuscripts must include a data availability statement. This statement should provide the following information, where applicable:

- Accession codes, unique identifiers, or web links for publicly available datasets
- A description of any restrictions on data availability
- For clinical datasets or third party data, please ensure that the statement adheres to our policy

Trial data can be made available by contacting the individual trial investigators of the trials included in this project, pending individual sponsors approval. The corresponding author of the paper can redirect requests. A specific data transfer agreement between the individual sponsors of the trials and the researcher may be requested. This data transfer agreement details both parts responsibilities to ensure the required level of data integrity and legal and ethical obligations.

Field-specific reporting

Please select the one below that is the best fit for your research. If you are not sure, read the appropriate sections before making your selection.

☒ Life sciences ☐ Behavioural & social sciences ☐ Ecological, evolutionary & environmental sciences

For a reference copy of the document with all sections, see nature.com/documents/nr-reporting-summary-flat.pdf

Life sciences study design

All studies must disclose on these points even when the disclosure is negative.

Sample size	Outlined in primary paper of this project : Loi et al J Clin Oncol. 2019 Mar 1;37(7):559-569.
Data exclusions	see Loi et al J Clin Oncol. 2019 Mar 1;37(7):559-569.
Replication	In some of the included studies in this pooled analysis, different pathologists have read TILs. The reproducibility of TILs reading has been adressed in other publications of the International TILs working group.
Randomization	Not relevant
Blinding	TILs reading was performed by pathologists without access to the clinical database.

Reporting for specific materials, systems and methods

We require information from authors about some types of materials, experimental systems and methods used in many studies. Here, indicate whether each material, system or method listed is relevant to your study. If you are not sure if a list item applies to your research, read the appropriate section before selecting a response.

Materials & experimental systems

n/a	Involved in the study	
☒	☐	Antibodies
☒	☐	Eukaryotic cell lines
☒	☐	Palaeontology and archaeology
☒	☐	Animals and other organisms
☐	☒	Human research participants
☐	☒	Clinical data
☒	☐	Dual use research of concern

Methods

n/a	Involved in the study	
☒	☐	ChIP-seq
☒	☐	Flow cytometry
☒	☐	MRI-based neuroimaging

Human research participants

Policy information about studies involving human research participants

Population characteristics	Pooled analyais of multiple individual studies. See Loi et al J Clin Oncol. 2019 Mar 1;37(7):559-569 for description.
Recruitment	See Loi et al J Clin Oncol. 2019 Mar 1;37(7):559-569 for description of inclusion periods.
Ethics oversight	Following french regulations, see Loi et al J Clin Oncol. 2019 Mar 1;37(7):559-569.

Note that full information on the approval of the study protocol must also be provided in the manuscript.

Clinical data

Policy information about clinical studies

All manuscripts should comply with the ICMJE guidelines for publication of clinical research and a completed CONSORT checklist must be included with all submissions.

Clinical trial registration	This project is not a clinical trial in itself.
Study protocol	Can be requested to the corresponding author.
Data collection	See Loi et al J Clin Oncol. 2019 Mar 1;37(7):559-569 for description.
Outcomes	See Loi et al J Clin Oncol. 2019 Mar 1;37(7):559-569 for description.
